# Impact of self-coiling catheters for continuous popliteal sciatic block on postoperative pain level and dislocation rate: a randomized controlled trial

**DOI:** 10.1186/s12871-022-01700-x

**Published:** 2022-05-24

**Authors:** Rosa Nickl, Oliver Vicent, Thomas Müller, Anne Osmers, Konrad Schubert, Thea Koch, Torsten Richter

**Affiliations:** 1 Department of Anesthesiology and Critical Care Medicine, University Hospital Carl Gustav Carus Dresden, Technische Universität Dresden, Fetscherstr 74, 01307 Dresden, Germany; 2 Institute for Medical Informatics and Biometry, Medical Faculty Carl Gustav Carus, Technische Universität Dresden, Fetscherstr 74, 01307 Dresden, Germany

**Keywords:** Perineural catheter, Popliteal sciatic block, Self-coiling catheter, Dislocation, Ultrasound- guided regional anesthesia

## Abstract

**Background:**

Dislocation of catheters within the tissue is a challenge in continuous regional anesthesia. A novel self-coiling catheter design is available and has demonstrated a lower dislocation rate in a cadaver model. The dislocation rate and effect on postoperative pain of these catheters in vivo has yet to be determined and were the subjects of this investigation.

**Methods:**

After ethics committee approval 140 patients undergoing elective distal lower limb surgery were enrolled in this prospective randomized controlled trial. Preoperatively, patients were randomly assigned and received either the conventional (*n* = 70) or self-coiling catheter (*n* = 70) for ultrasound-guided popliteal sciatic nerve block in short axis view and by the in-plane approach from lateral to medial. The primary outcome was pain intensity after surgery and on the following three postoperative days. Secondary outcomes investigated were dislocation rate in situ determined by sonography, catheter movement visible from outside, opioid consumption as well as leakage at the puncture site.

**Results:**

All catheters were successfully inserted. The study population of self-coiling catheters had significantly lower mean numeric rating scale values than the reference cohort on the first (*p* = 0.01) and second postoperative days (*p* < 0.01). Sonographic evaluation demonstrated, 42 standard catheters (60%) and 10 self-coiling catheters (14.3%) were dislocated in situ within the first three postoperative days. The externally visible movement of the catheters at insertion site did not differ significantly between groups through the third postoperative day. The opioid consumption was significantly lower in the self-coiling catheter group on the day of surgery and on the second and third postoperative days (*p* = 0.04, *p* = 0.03 and *p* = 0.04, respectively).

**Conclusion:**

The self-coiling catheter offers a better postoperative pain control and a lower dislocation rate within the tissue when blocking the popliteal sciatic nerve compared to a conventional catheter. Further trials in large patient cohorts are warranted to investigate the potential beneficial effects of self-coiling catheters for other localisations and other application techniques.

**Trial registration:**

The trial was registered at German Clinical Trials Register (DRKS) on 08/04/2020 (DRKS00020938, retrospectively registered).

## Background

Continuous nerve blocks play an essential role in modern multimodal analgesia concepts [1]. Regional anesthesia is widely used in orthopaedic and trauma surgery on the lower limb [2]. For both the lower and upper extremities, randomized controlled trials have shown significant pain reduction with continuous regional anesthesia after surgery [3, 4]. This also leads to less chronic pain, a reduced need for opioids and fewer associated side effects such as nausea, vomiting, constipation and fatigue [3, 5]. However, some studies have found that the analgesic benefits of continuous regional anesthesia fade after 24- 48 h [6, 7]. The secondary failure rate of indwelling catheters for regional anesthesia has been reported in the literature to be as high as 40% [8, 9]. In addition to initial misplacement secondary catheter dislocation in the postoperative course could be a possible explanation for the worsened efficacy of continuous regional anesthesia [10]. Secondary dislocation is commonly defined as outwardly visible displacement or sliding out of the catheter, at times inadvertently as a result of patient movement [11, 12]. However, regarding dislocations, the rare event of external dislodgement at the insertion site has to be distinguished from internal catheter tip migration away from target structure or nerve surrounding compartment due to active and passive movement of adjacent muscles. The latter problem might be underestimated since pain scores, opioid consumption, sensory block distribution and patient satisfaction serve only unreliable surrogate measures for correct catheter position. Investigation of internal dislocations by direct visualization of the catheter tip or better by imaging of fluid spread referred to the nerve has been addressed only in a few studies with limited number of patients [10, 13, 14]. Two more studies have investigated dislocation rate in situ, either solely in healthy volunteers [15] or in human cadavers [16]. Stiff catheters placement using the most popular short axis (SAX) / in-plane (IP) approach might bear an increased risk of internal dislocations [10, 16] since the catheter mostly can be placed only a short distance beyond the needle tip to avoid bypassing the nerve [17].

A new catheter design with a 2.5 cm long self-coiling soft end that remains close to the nerve that has shown a very low risk of initial misplacement in cadavers for paravertebral block and sciatic nerve block [18, 19]. Dislocation in vivo may be due to patient movement and deserves therefore special attention during catheter evaluation. However, so far, no studies have investigated if self-coiling catheters are also more resistant to secondary dislocations with progressive improvement of pain management in surgical patients.

We compared the self-coiling catheter with regular straight catheters for continuous popliteal sciatic blockade regarding analgesic efficacy and position change within and outside the tissue.

## Methods

### Study design

This study was a prospective, randomized controlled, single-centre trial.

### Ethics

Positive ethic votum was approved by the Institutional Review Board of the Technische Universität Dresden (EK150042016). Written informed consent was obtained from all patients. This study is registered at the German Clinical Trials Register (DRKS00020938) and is reported according to the CONSORT guidelines 2010 [20].

### Patients and randomisation

**Fig. 1:**
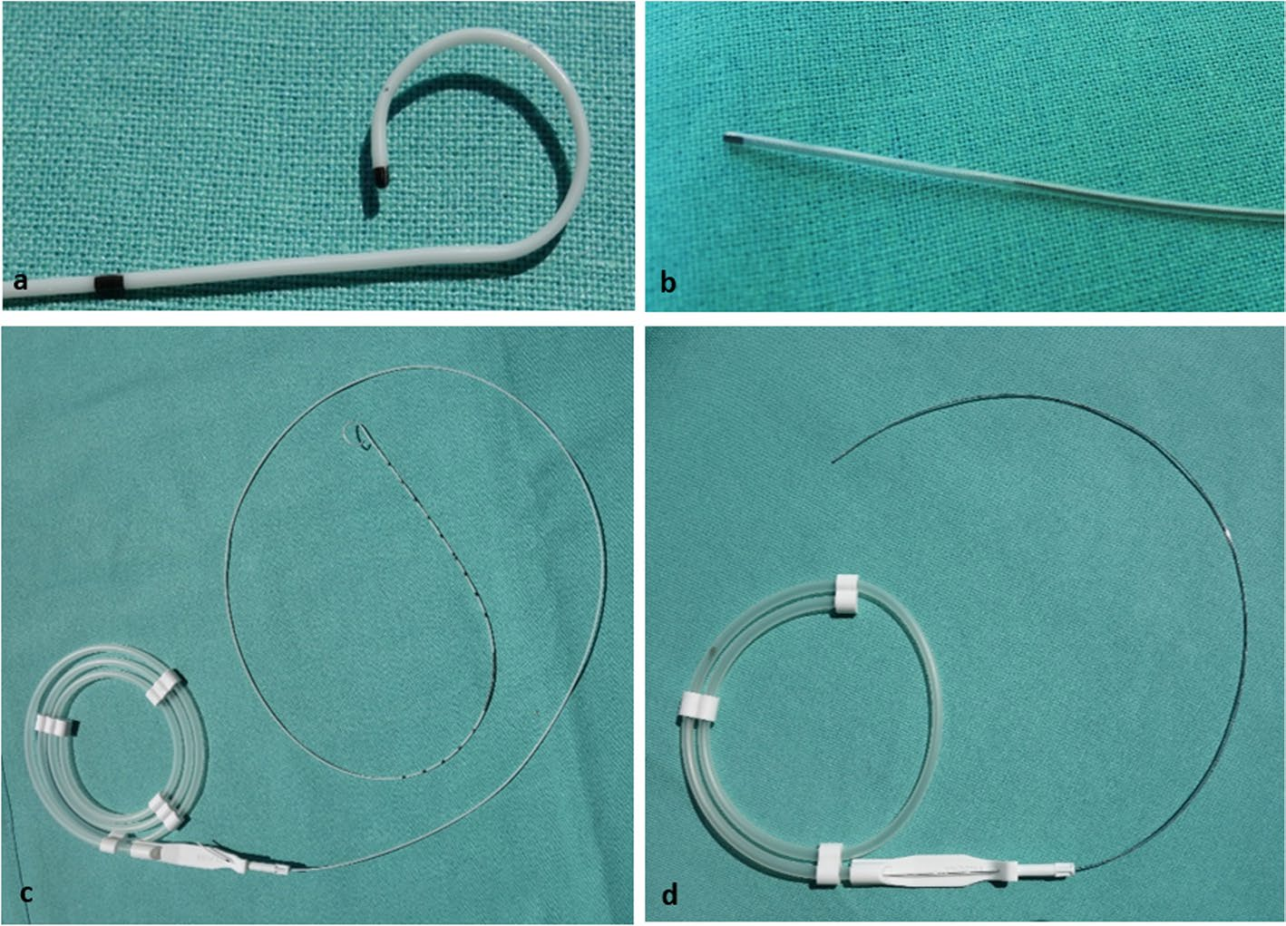
Study catheters and needle. **a **+** c** Self-Coiling Catheter (Sono Long Curl Echo 20 G 100 mm, Pajunk medical products, Geisingen, Germany), **a** catheter tip; **c** entire catheter. **b **+** d** Regular straight catheter (Sono Long Sono 20 G 100 mm, Pajunk medical products, Geisingen, Germany), **b** catheter tip; **d** entire catheter

140 adult patients scheduled for continuous regional anesthesia with popliteal sciatic catheter as part of elective major ankle or foot surgery at the University Hospital Carl Gustav Carus at the Technische Universität Dresden, were enrolled between 09/2016 and 12/2017 for this trial. The applied inclusion and exclusion criteria applied are summarized in Table 1. Randomisation was carried out immediately before catheter insertion by means of sequentially numbered sealed opaque envelopes, containing the study number and corresponding reference to the respective group. Patients were assigned to two groups: an interventional group, in which the self-coiling catheter (SCC, SonoLong Curl Echo 20 G 100 mm, Pajunk medical products, Geisingen, Germany) was inserted and a control group, which received the regular straight catheter (RSC, SonoLong Sono 20 G 100 mm, Pajunk medical products, Geisingen, Germany) for popliteal sciatic block (Fig. 1). Surgeons, nurses, patients and members of the acute pain service, as well as the investigators, with the exception of the anesthesiologists who inserted the catheter, were blinded to the study groups. Investigators who collected the data were not blinded to the groups.

**Table 1:** Inclusion and exclusion criteria

Inclusion criteria	Exclusion criteria
age between 18 and 75 years indication for the application of a distal sciatic catheter within the scope of an elective surgical procedure patient consent	patient diagnosed with chronic pain prior to surgery polyneuropathy or ipsilateral neuropathy, involving the lower limb refusal of regional anesthesia patient not legally competent intolerance or allergy to ropivacaine or oxy-codone neuromuscular diseases BMI > 35 kg/m.^2^ pre-existing opioid medication due to the injury to be operated on

### Application of regional anesthesia

Patients were placed in supine position with the relevant leg elevated. After generous skin disinfection (ChloraPrep, Becton, Dickinson and Company, Franklin Lakes, USA) the area was covered with a sterile fenestrated sheet. The transducer was draped with a sterile ultrasound probe cover (CIV-Flex® Transducer Covers, Civco Medical Solutions, Kalona, Iowa, USA).The sciatic nerve was visualized from the posterior thigh in short axis view (SAX) at the level of the popliteal nerve bifurcation with a 4–12 MHz linear probe (L12-4) of a Philips Sparq or Philips Affiniti 70G ultrasound system (Philips Healthcare, Andover, Massachusetts, USA) or 5- 13 MHz linear probe (12L-RS) of a GE Logiq e ultrasound system (GE Healthcare, Milwaukee, Wisconsin, USA). Nerve bifurcation was defined as the most proximal point where the tibial and common fibular nerve clearly started to bifurcate. After skin infiltration with 2–4 ml prilocaine 1%, an 18 G Tuohy needle was advanced using an in-plane technique from lateral until the needle tip was located within the paraneural sheath. The designation of the sheaths of the sciatic nerve is referred to in the previous publication of Andersen and colleagues [21]. Special care was taken not to touch the nerve or puncture the epineurium. An initial bolus of 20 ml ropivacaine 0.5% (Naropin 10 mg/ml, Astra Zeneca, London, UK) was applied directly via the injection line connected to the Tuohy puncture cannula under direct sonographic visualization. Local anesthetic spread was observed circumferentially around both components of sciatic nerve within the paraneural sheath. If diffuse spreading into the surrounding tissue occurred, the cannula position was corrected. Afterwards, the previously randomized catheter was placed adjacent to the sciatic nerve within the paraneural sheath through the cannula. According to manufacturer instructions SCC was advanced approximately 2.5–3 cm beyond the needle tip to facilitate the coiling up of the distal end of the catheter end. The RSC was inserted approximately 3–4 cm beyond the needle tip. Proper position of the catheter was then confirmed sonographically by observing the spread of a 2 ml bolus of saline injected into the paraneural sheath via the catheter. In case of spreading outside the paraneural sheath, the catheter was retracted under real-time sonographic guidance until the injected saline bolus was reliably distributed around the nerves. The catheter was then connected to the associated bacterial filter and fixed with sterile suture strips (Omnistrip®, Fa. Paul Hartmann AG, Heidenheim, Germany). Finally, a sterile film dressing (IV3000 10 × 12 cm, Smith & Nephew Medical Ltd., London, UK) was used to additionally fix the catheter. All catheters were placed as a part of the clinical anesthesia routine by a total of four senior anesthesiologists who have extensive experience with ultrasound-guided blockade of popliteal sciatic nerve. The success of the sciatic blockade was evaluated in all patients by testing warm-cold differentiation in the innervation area.

### Additional anesthetic procedures and hemodynamic monitoring

**Fig. 2:**
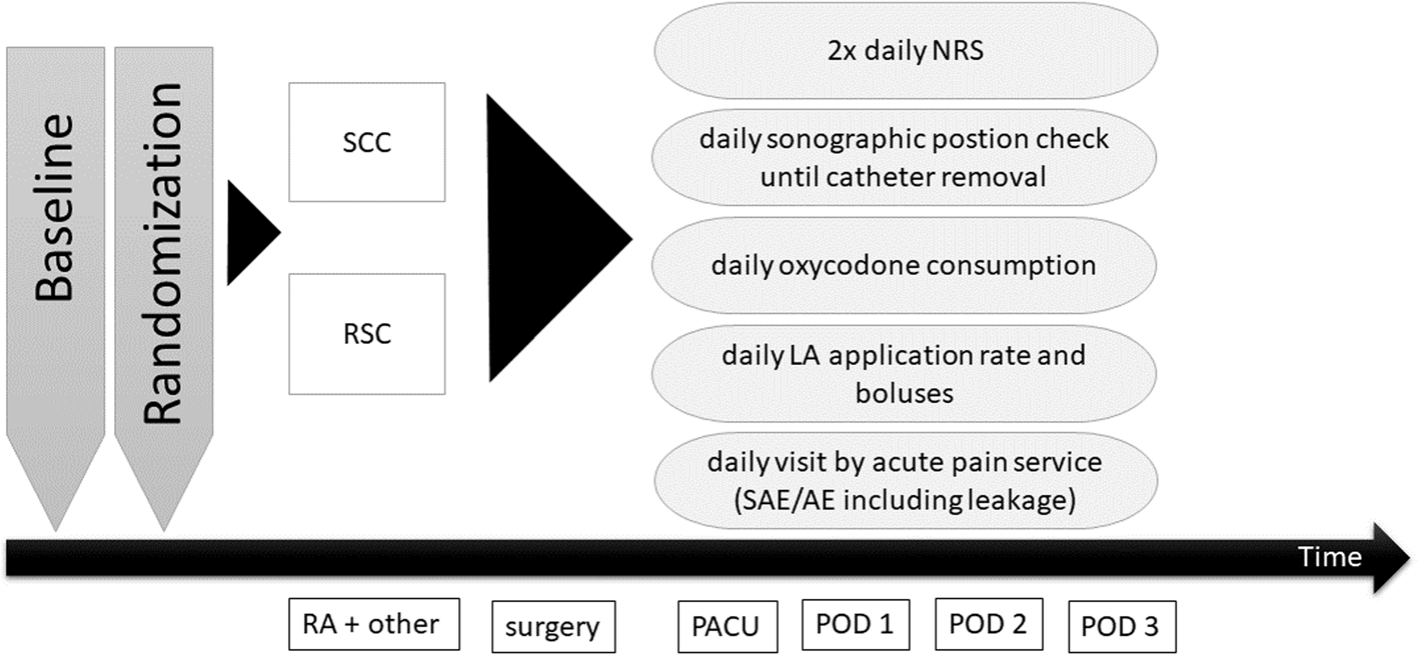
Time course of proceeded interventions. Baseline characteristics were assessed as shown in Table 2. *AE* adverse events, *NRS* numeric rating scale, *PACU* postoperative anesthesia care unit, *POD* postoperative day, *RA* regional anesthesia, *RSC* regular straight catheter group, *SAE* severe adverse events, *SCC* self-coiling catheter group

The time course of proceeded interventions were summarized in Fig. 2. After arriving in the operating area, peripheral venous access with an infusion of a balanced crystalloid solution, a 3- or 5-lead ECG monitoring including ST-segment analysis, a pulsoxymetry and non-invasive blood pressure measurement were established. Hemodynamic data were continuously recorded using a Philips Intellivue MP 70 (Philips Medicine Systems GmbH, Hamburg, Germany). If surgery involved the medial area of the lower leg, ankle or foot, an additional ultrasound guided saphenous nerve block was performed with 10 ml ropivacaine 0.5% (Naropin 10 mg/ml, Astra Zeneca, London, UK) via an 80 mm 22 G Sonoplex cannula (Pajunk medical products, Geisingen, Germany) at femoral triangle by SAX view and in-plane approach. Further anesthetic procedures were based on the individual risk profile, the patient´s comfort level and the requirements of planned surgical procedures, such as the use of thigh tourniquet. Femoral nerve block was performed together with an obturator nerve block when a tourniquet at the thigh was required and an additional spinal or general anaesthesia was to be avoided. Regardless of randomization, in addition to the continuous peripheral sciatic blockade the following procedures were used in addition to the continuous peripheral sciatic blockade: anesthesia standby (no further intervention, anaesthesiologist on site to monitor vital functions during surgery), sedation, femoral and obturator nerve block and spinal or general anesthesia. For spinal anesthesia 2 to 2.4 ml of hyperbaric bupivacaine 0.5% (Bucain hyperbar 5 mg/ml, PUREN Pharma GmbH & Co. KG, Munich, Germany) with 10 µg fentanyl via a 25 G Sprotte cannula (Pajunk medical products, Geisingen, Germany) was used. Sedation was applied by use of propofol 20 mg/ml (Fresenius Kabi Deutschland GmbH, Bad Homburg, Germany) with a rate of 1–2 mg/kg/h. General anesthesia was induced and maintained with propofol (Propofol 1% and Propofol 2%, Fresenius Kabi Deutschland GmbH, Bad Homburg, Germany) and sufentanile (Sufentanil-hameln 5 µg/ml, hameln pharmaceuticals gmbh, Hameln, Germany). The airway was secured with a laryngeal mask (Ambu® AuraGain™, Ambu GmbH, Bad Nauheim, Germany).

### Postoperative procedure and outcome parameter assessment

After surgery, the patients were observed under cardiovascular monitoring in the post anesthesia care unit, if necessary. They were then transferred to the general ward. Postoperatively, both groups received continuous application of ropivacaine 0.2% (Naropin 2 mg/ml, Astra Zeneca, London UK) at an initial rate of 6 ml/h. As part of a multimodal pain management approach, all patients received oral ibuprofen 600 mg every eight hours as a baseline analgesic. In case of contraindications to ibuprofen, novamine sulfone (1 g every six hours) was administered alternatively per os or intravenously. The standard multimodal analgesic regimen with novamine sulfone or ibuprofen at the above-mentioned dosages, was maintained postoperatively. Patients were visited regularly twice daily by the acute pain service beginning on the first post-operative day (POD). Pain service visit included monitoring for signs of infection, leakage and external dislocation at insertion site. In order the numerical rating scale (NRS 0–10) was used twice daily as a semi-quantitative method to assess the patient´s subjective pain intensity. The first interview was conducted two hours after the end of surgery. The NRS, the spread of the sciatic block, and any adverse events were recorded. For rescue pain relief (NRS > 3), either a manual bolus of 10 ml ropivacaine 0,3% (Naropin, Astra Zeneca, London, UK) could be administered by the acute pain service staff or patients received oxycodone 10 mg (Oxygesic akut 10 mg, Mundipharma GmbH, Limburg, Germany) per os from the ward nurses. The total daily ropivacaine requirements of ropivacaine and total oxycodone consumption were recorded.

**Fig. 3:**
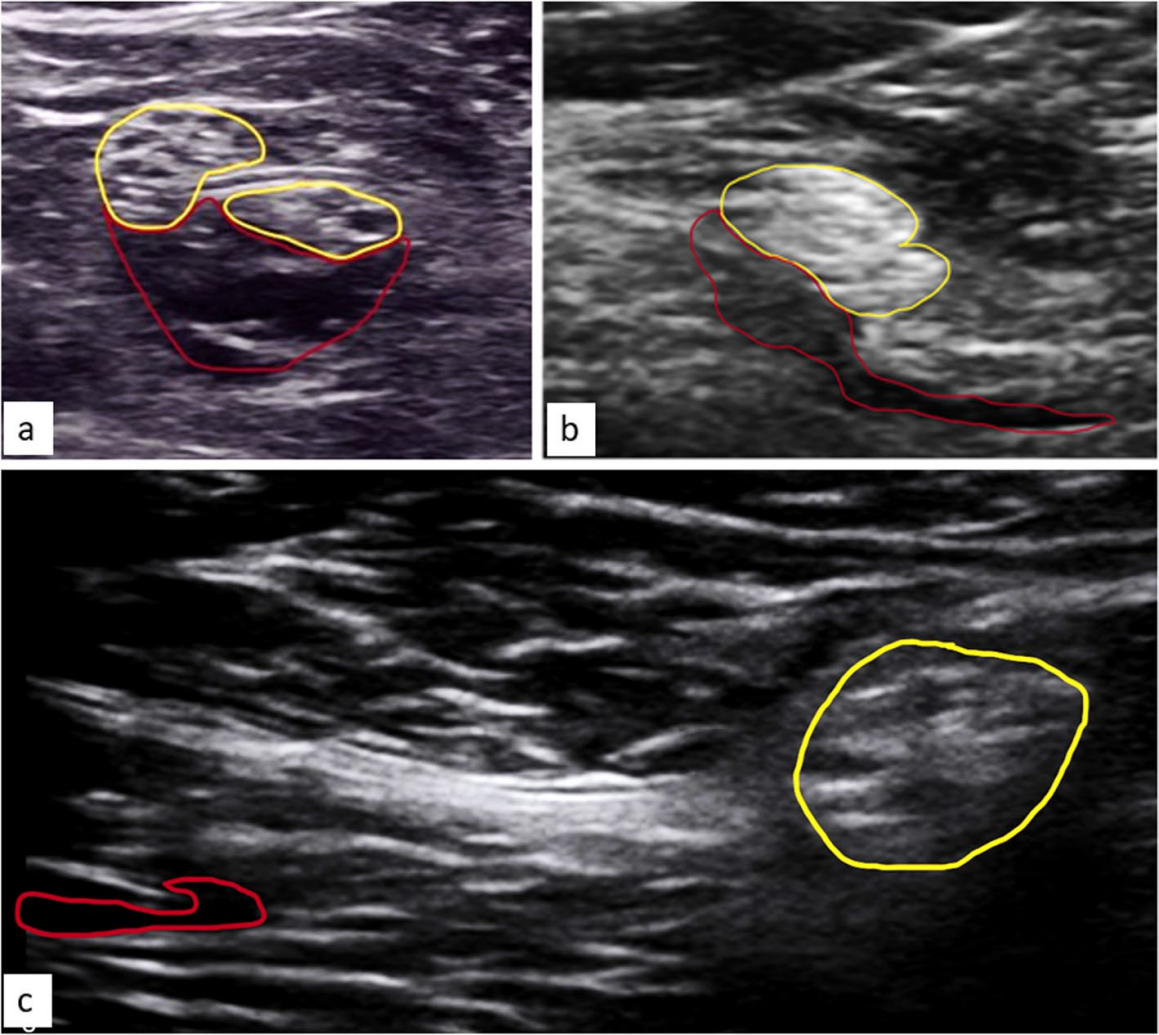
Catheter position categories in the sonographic image. **a**- perineural intrafascial position: catheter tip is located within the paraneural sheath indicated by saline bolus spread. **b**- 1^st^ degree dislocation, also called perineural extrafascial: catheter tip is located outside the paraneural sheath but still close to the nerve. Saline bolus (red frame line) moisture the fascial paraneural sheath (yellow frame line) but run away into surrounding tissue. This category is also recorded as dislocated for evaluation since efficacy is decreased. **c**- 2^nd^ degree dislocation, catheter tip is outside the paraneural sheath without proximity to the nerve. Saline bolus spreads diffuse within the biceps femoris muscle. White arrow- catheter, * preferred correct position

The position of indwelling catheters was identified immediately after surgery at the PACU and daily on the first, second and third POD. For this purpose, we connected a syringe containing 6 ml of saline to the proximal end of the catheter. We then injected 2 ml of saline and monitored its distribution at the distal end of the catheter in the tissue under sonographic view. If the distribution of the saline could not be seen on the first attempt, we repeated the procedure one or two times until the distribution could be imaged. The distribution of the saline in relation to the nerve and the adjacent paraneural sheath was evaluated as a surrogate for the position of the distal end of the catheter. Corresponding images were digitally stored and the determined position was documented. Three different positions of the catheter in relation to the nerve were categorized, as shown in Fig. 3. In case of catheter dislocation, no further US-guided examinations were subsequently performed.

### Safety thresholds

Participation in the study could be terminated at any time, if any of the following dropout criteria were met: patient´s request, allergic reaction to ropivacaine, novamine sulfone or ibuprofen, need for second surgery during the follow-up period.

### Primary and secondary outcomes

The primary outcome was the individual postoperative pain intensity, assessed with the numerical rating scale (NRS) after surgery on the lower leg or foot in the first 72 h. The patient rated the pain on a scale from 0 to 10. Recorded pain scores recorded were averaged per day per patient. The following issues were identified as secondary outcomes: additional need for systemic opioids, rate of secondary dislocations within the tissue and dislocations and leakage at the injection site.

### Statistical analysis

The sample size was calculated as followed. To demonstrate a clinically relevant difference of 1 level on an NRS of 0 to 10, at least 64 patients were enrolled with a standard deviation of 2, a significance level of 0.05 and a power of 80%. A drop-out rate of 8% was expected. Therefore, 70 patients per group were included. According to the sample size design, the analysis was carried out with an unpaired two-sided Student´s t-test at 5% level assuming equality of variance. The distribution of continuous parameters was described using of mean value and standard deviation (normally distributed) or by medians and quartiles. Categorical parameters were described by their absolute and relative frequencies, and differences between groups were examined with Pearsons´s chi- square test or Fisher´s exact test. Two-group comparisons of metrically scaled variables were performed with independent two-tailed t-tests after checking equality of variances with Levene´s test. To analyse the influence of possible covariates of the respective baseline data, an analysis of covariance was calculated. For correlation of nominal and metric parameters, eta correlation coefficient was calculated. Statistical significance was considered at two-sided *p* < 0.05.

All calculations and graphs were performed and computed using SPSS (IBM SPSS Statistics Vers. 25, IBM Deutschland GmbH, Ehningen, Germany).

## Results

**Fig. 4:**
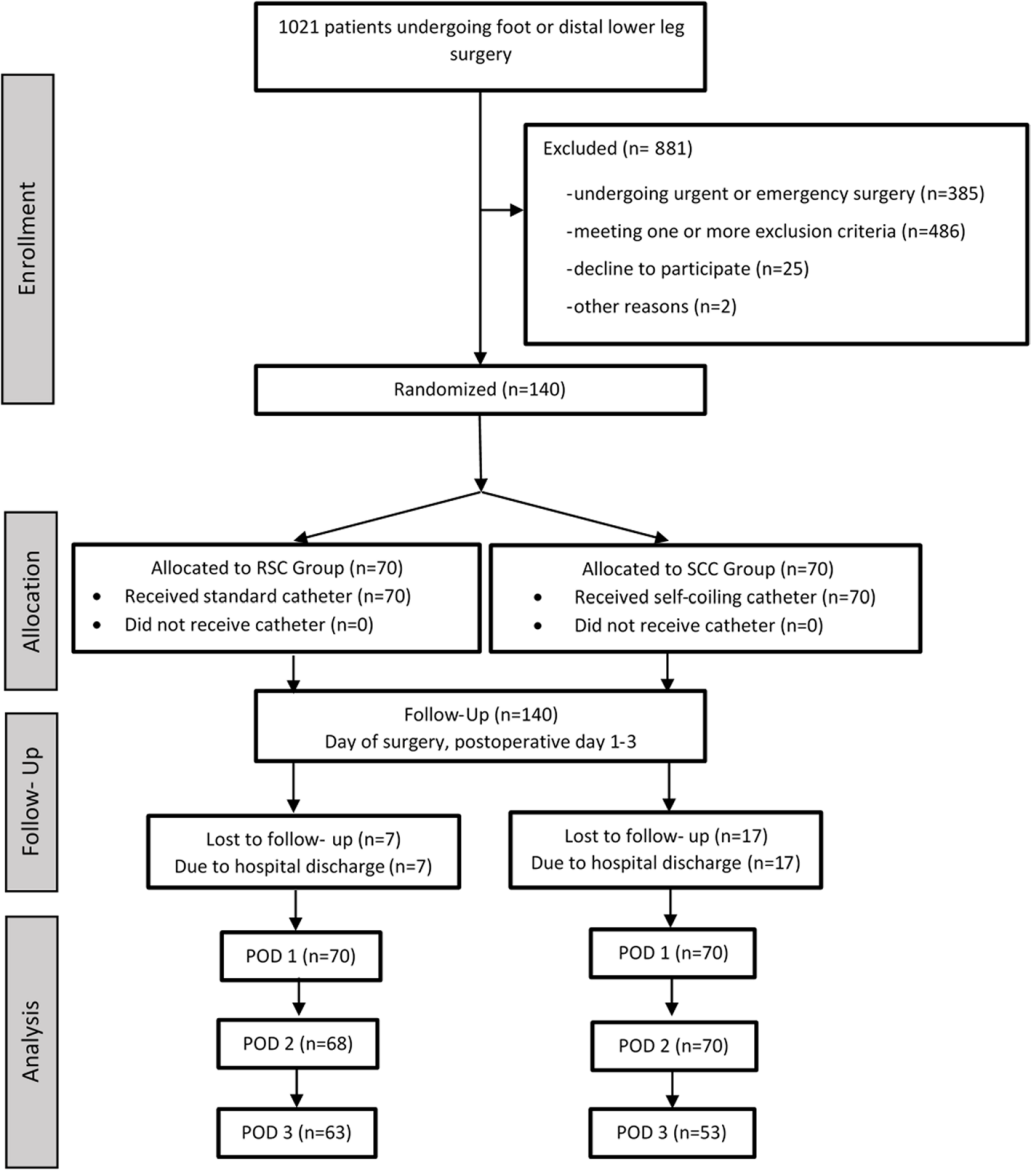
Flow chart. 140 consecutive patients were enrolled in this trial. Patients got lost to follow-up only because of earlier hospital discharge. No patient has withdrawn his consent. *POD* postoperative day, *RSC* regular straight catheter, *SCC* self-coiling catheter

From 09/2016 to 12/2017 1021 patients undergoing elective ankle or foot surgery were screened and. 140 patients were included in this study (Fig. 4). Both groups were comparable in terms of baseline. characteristics as depicted in Table 2. The study was closed after inclusion of the planned 140 patients.

**Table 2:** Baseline characteristics

	RSC Group (*n* = 70)	SCC Group (*n* = 70)
Age [years]	50 ± 14	50 ± 13
Gender [no/%]		
Female	37 (53)	37 (53)
Male	33 (47)	33 (47)
Body height [cm]	171.7 ± 9,0	173.1 ± 10.2
Body weight [kg]	76.5 ± 13.7	79.3 ± 16.5
BMI [kg/m.^2^]	25.9 ± 3.7	26.3 ± 4.3
ASA [no./%]		
I	38 (54)	35 (50)
II	27 (39)	33(47)
III	5 (7)	2 (3)
Surgical area		
Upper ankle joint	35 (50)	41 (59)
Calcaneus	8 (11)	14 (20)
Hallux	10 (14)	6 (9)
Talus	6 (9)	0 (0)
Metatarsal	10 (14.3)	7 (10)
Tendons/syndesmosisof the ankle	1 (1)	2 (3)

Values are given as absolute number (percentage) or mean (± standard deviation), as appropriate. ASA American Society of Anesthesiology physical status, *BMI* Body mass index, *RSC* regular straight catheter, *SCC* self-coiling catheter

### Catheter placement and additional anesthetic procedures

Sonographic identification of the nerve and bifurcation was successful in all patients. The mean time required for catheter placement was 12.9 ± 4.3 min in both study groups (*p* = 0.97). Catheter placement was successful in all patients, as indicated by correct distribution of local anesthetic within the paraneural sheath of the sciatic nerve and complete loss of sensory and motoric function in the supplied nerve area. As shown in Table 3, there were no statistic significant differences with respect to the duration of surgery and initial amount of ropivacaine administered. No differences were found between the groups with regard to the number of additional saphenous nerve blocks and other additional anesthetic procedures or the usage of postoperative basic analgesics.

**Table 3:** Additional anesthetic procedures, duration of catheter placement and surgery, postoperative basic analgesic medication, ropivacaine consumption and leakage

	**RSC Group**	**SCC Group**	***P* Value**
**Saphenous nerve block**	64 (91.4)	56 (80)	0.05
**Additional anesthetic procedure [no/%]**			0.17
General anesthesia	53 (75.7)	60 (85.7)	
Spinal anesthesia	10 (14.3)	6 (8.6)	
Femoral nerve and obturator nerve block	0 (0)	2 (2.9)	
Analgosedation	6 (8.6)	2 (2.9)	
none	1(1.4)	0 (0)	
**Duration of catheter placement [min]**	12.9 ± 4.3	12.9 ± 4.3	0.97
**Duration of surgery [min]**	94.4 ± 41.0	96.2 ± 39.4	0.79
**Postop. analgesic medication [no/%]**			0.2
Ibuprofen	46 (65.7)	55 (78.6)	
Novamine sulfone	19 (27.1)	13 (18.6)	
Ibuprofen + Novamine sulfone	5 (7.1)	2 (2.9)	
**Ropivacaine consumption [mg/ 24 h]**			
POD 0	283.8 ± 34.7	288.7 ± 10.0	0.26
POD 1	264 ± 112.1	233 ± 101.5	0.09
POD 2	108 ± 124.9	127.5 ± 116.7	0.38
POD 3	23 ± 84.4	52.1 ± 93.4	0.22
**Leakage [no/%]**			
POD 0	8 (11.4)	6 (8.6)	0.83
POD 1	22 (31.4)	21 (30)	0.84
POD 2	21 (30)	22 (31.4)	0.6
POD 3	3 (4.3)	9 (12.9)	0.07

Values are given as absolute number (percentage) or mean (± standard deviation), as appropriate. Difference between groups were tested with Chi-square test or two-sided Student´s t-test with statistical significance considered at *p* < 0.05, *POD* postoperative day, *RSC* regular straight catheter, *SCC* self-coiling catheter

### Primary outcome parameter- postoperative pain level

**Fig. 5:**
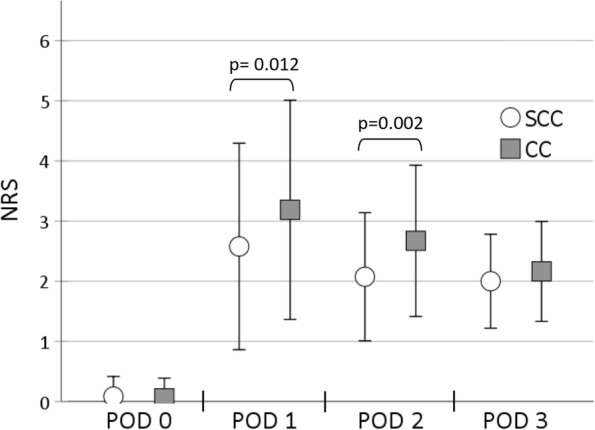
Postoperative pain scores. Values are given as mean ± standard deviation on numeric rating scale. Statistical significance was considered to be at two-sided *p* < 0.05. Differences between groups were analysed using Student´s t-test. *NRS* numeric rating scale*, POD* postoperative day, *RSC* regular straight catheter, *SCC* self-coiling catheter

Pain scores recorded daily over the observation period are shown in Fig. 5. The NRS values did not differ significantly on the day of surgery (*p* = 0.69). On POD 1 and POD 2, patients in the SCC group had significantly less pain than those in the RSC group. On POD 3, there was no difference in pain intensity between the two study groups.

### Secondary outcome parameters

#### Opioid consumption

The determined values of oxycodone consumption show a large scatter and are summarized in Table 4. All mean values of the oxycodone consumption are lower with the self-coiling catheter than with the conventional catheter. Significant differences between the two groups were found on the day of surgery (*p* = 0.04), POD 2 (*p* = 0.03) and POD 3 (*p* = 0.04). For POD 1 (*p* = 0.19) a tendency in lower oxycodone consumption was observed.

**Table 4:** Oxycodone consumption of both study groups during study period

	RSC Group	SCC Group	*P* Value
Daily Oxycodone consumption (mg)			
POD 0	1.29 ± 4.45	0.14 ± 1.19	0.04*
POD 1	15.57 ± 19.16	11.71 ± 15.32	0.19
POD 2	12.50 ± 14.90	7.57 ± 11.60	0.03*
POD 3	7.94 ± 8.64	4.92 ± 7.88	0.04*

Values are given as mean (± standard deviation). Statistical significance considered at *p* < 0.05 (*). *POD* postoperative day, *RSC* regular straight catheter, *SCC* self-coiling catheter

#### Dislocation

**Fig. 6:**
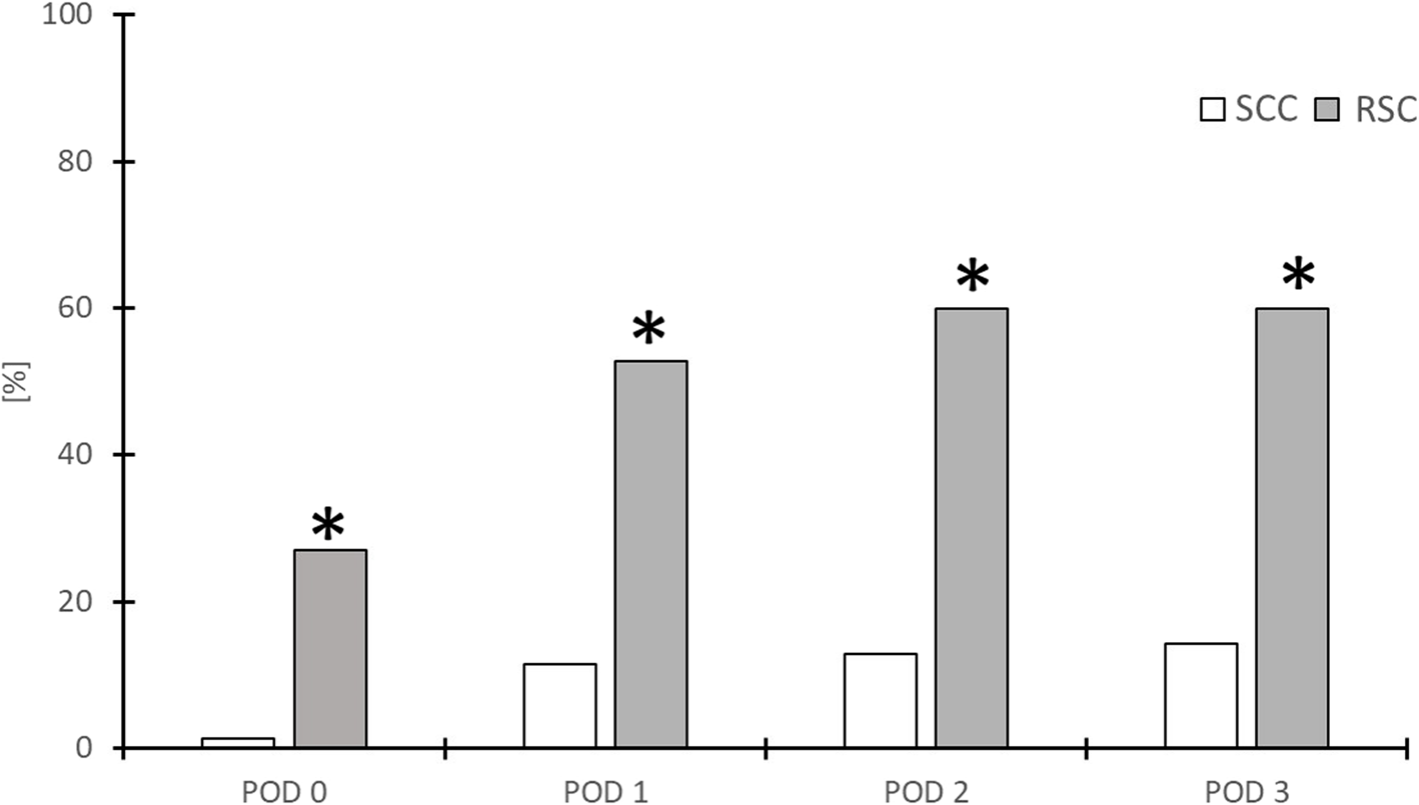
Percentual cumulative dislocation rate of the catheters. A Chi-square test with multiple regression approach was performed. Statistical significance was accepted at *p* < 0.05. *POD* postoperative day, *RSC* regular straight catheter, *SCC* self-coiling catheter, * *p* < 0.05

**Fig. 7:**
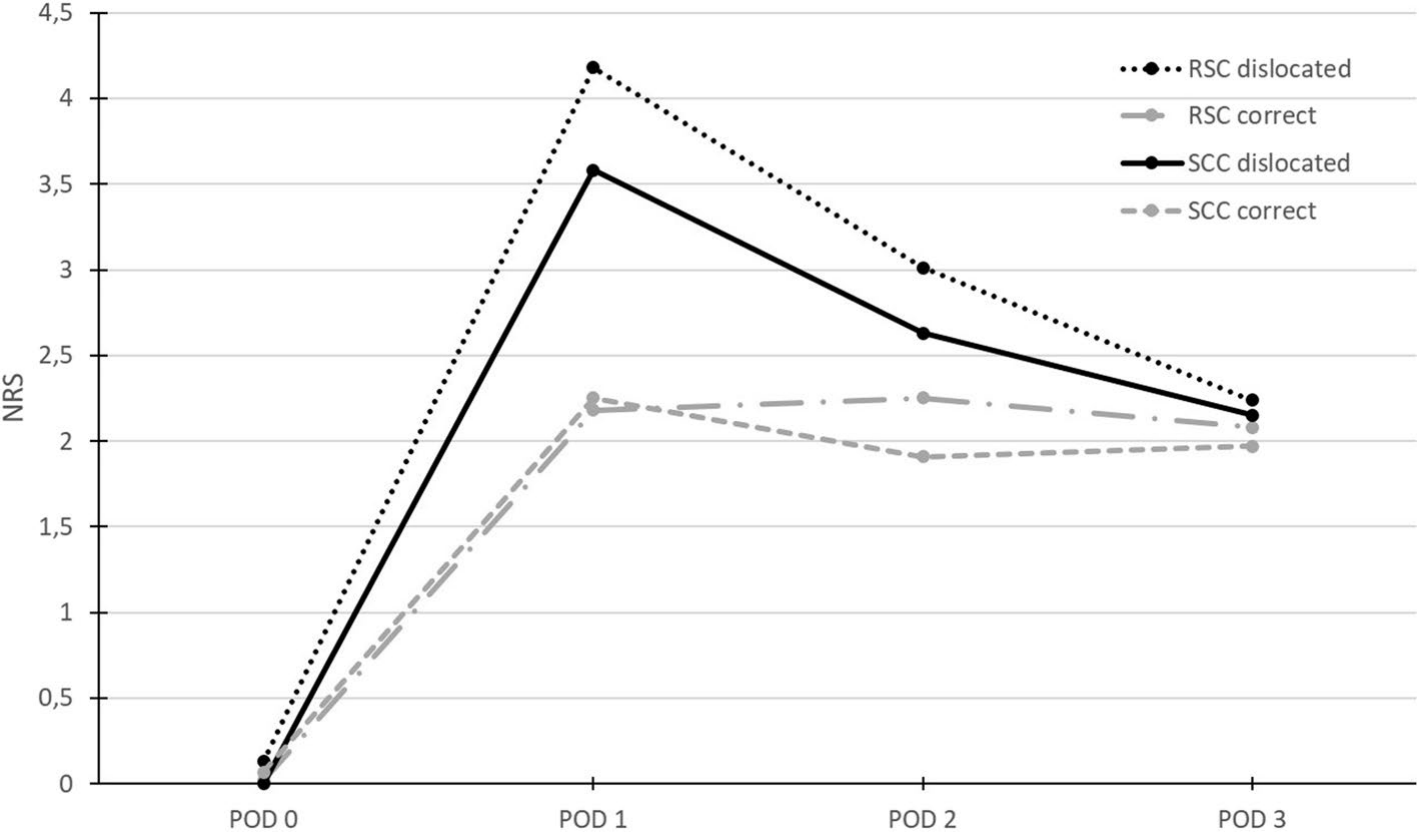
Mean values of NRS assessment as a function of time for the different catheter types and dislocation categories in situ. *NRS* numeric rating scale, *POD* postoperative day, *RSC* regular straight catheter, *SCC* self-coiling catheter

The spread of fluid was demonstrated in all sonographic examinations performed throughout the study. The dislocation rates are shown in Fig. 6. The majority of dislocations were detected after the surgical procedure and transfer of the patient to the PACU. However, catheter dislocation was significantly lower in the SCC group. (RSC *n* = 19 vs. SCC *n* = 1, *p* < 0.01). Over the entire observation period, catheters in the SCC group dislocated less frequently than in the RSC group (*p* < 0.01 for POD 1 & 2, *p* = 0.01 for POD 3, respectively). The cumulative dislocation rate was 60% (*n* = 42) in RSC group and 14.3% (*n* = 10) in SCC group. A significant influence of dislocation on the mean indicated NRS value was demonstrated for POD 1 and POD 2 (POD 1 *p* < 0.01; POD 2 *p* < 0.01), as shown in Fig. 7. Fluid distributed through all nondislocated catheters was perineurally at the bifurcation of the sciatic nerve, whereas fluid distribution in dislocated catheters was generally lateral to the nerve branch along the original needle track.

#### Catheter insertion depth

The puncture depth of the two catheter groups was not significantly different (*p* = 0.169) and averaged 5.8 (RSC) and 6.0 cm (SCC). However, the distance between the puncture depth and the final skin level of the catheters differed due to catheter design. The self-coiling catheters were placed at a mean of 2.4 cm above the needle tip position. For the regular catheters, this value was 1.6 cm (*p* < 0.01). No catheter in either group with an insertion depth greater than/equal to 3.5 cm dislocated in this study. The eta coefficient was 0.380 (*p* < 0.01), giving a moderate correlation. Thus, 14.4% of the variance in dislocation can be explained by the insertion depth.

#### External movement at the insertion site

The externally visible movement of the pain catheters was documented daily. In contrast to the dislocation within the tissue, no significant difference between the two groups regarding the externally visible position at the insertion site could be detected over the entire observation period (POD 0, *p* = 0.86; POD 1, *p* = 0.39; POD 2, *p* = 0.65). Overall, 7 (10%) of the self- coiling catheters and 9 (12.9%) of the regular straight catheters slipped back at least 1 cm or further (*p* = 0.6). We could not detect any differences between correctly positioned and dislocated catheters in terms of catheter outward movement.

#### Leakage

Overall, puncture site leakage was most frequently observed on the first and second postoperative days. We observed no significant differences in characteristics between the SCC and RSC group (POD 0, *p* = 0.83; POD 1, *p* = 0.84; POD 2, *p* = 0.6; POD 3, *p* = 0.07) as seen in Table 3.

#### Complications due to perineural catheter

Eight patients (5.7%) experienced mild local inflammation at the insertion site, which resolved completely after removal during the remainder of the study. Due to catheter occlusion, one catheter in each study group (0.7%) could no longer be used on the first or second postoperative day. One patient in the RSC group complained of a metallic taste and nausea after the infusion rate of ropivacaine was increased from 6 to 8 ml/h Ropivacaine 0.2% and a bolus was administered. The catheter was then immediately removed and all symptoms were completely resolved within a few hours. No neurologic deficit occurred during the observation period. Catheter kinking did not occur in any of the study groups.

## Discussion

In this randomized controlled trial of continuous popliteal sciatic blocks, we demonstrated the superiority of self-coiling catheters over conventional straight catheters in terms of pain management and internal dislocation rate within the tissue. Leakage and external dislodgement of the catheter at the insertion site did not differ between the groups, nor did they contribute to dislocations in the tissue. The higher stability of the self-coiling catheter in its original positioning could result in less pain on the first and second day after surgery and less opioid consumption compared with patients in the RSC group. To our knowledge this is the first study to investigate the impact of different catheter characteristics on secondary dislocations and the benefits of a self-coiling catheter for peripheral nerve block in clinical practice.

### Pain intensity

We did not observe any NRS-differences between the two catheter types on the day of surgery. The dislocation rate on the day of operation did not seem to affect pain scores, because the initial bolus of ropivacaine 0.5% was equally effective in both groups and produced a long-lasting blockade of pain perception. Ropivacaine produces nerve blockade for up to 18 h, depending on dose and proximity of applied LA to the nerve. Christiansen et al. described a mean duration of action for distal sciatic blocks of more than 13 h with a lower dose of 60 mg ropivacaine [22]. In this study 100 mg per patient was administered initially. Several other studies have reported similar results. Some investigators compared a single popliteal blockade of the sciatic nerve with a continuous application of local anesthetic via catheter and found no significant differences in pain intensity on the day of surgery for either group [23–25]. However, pain level were significantly lower in the SCC group on the first and second POD. The difference of 0.7 points on NRS appears clinically insignificant, but may become relevant for the individual patients. The reason for the higher pain scores in the RSC group on POD 1 and 2 might be related to a higher dislocation rate, where the catheter tip comes to rest outside the nerve fascia. Thus, a lower effective dose of continuous infused ropivacaine could act on the sciatic nerve. In contrast, the lack of difference on POD 3 might be explained by spontaneous pain relief in the late postoperative period.

With decreasing effect of the initial bolus of 0.5% ropivacaine on the first postoperative day, the average pain level of all participants was the highest compared with the other measurement points. This could be caused by dislocations of indwelling catheters, inadequate lower continuous dose of ropivacaine and pain perception in the medial aspects of the ankle and foot not covered by continuous sciatic nerve block when the effect of saphenous or femoral nerve block had worn off [26]. In particular pain perception in sensory area of the saphenous nerve may have contributed to the convergence of pain intensity scores and the spread of differences between the groups in perineural sciatic nerve catheter performance. The use of an additional saphenous nerve catheter could possibly have reduced pain scores and additional opioid consumption. However, reports on the effects of the continuous or prolonged saphenous nerve blockade on pain after ankle surgery are conflicting [27–29].

Among nearly 71,000 patients undergoing 179 different surgeries in all body regions, the calcaneal surgery was found the most painful surgery with a mean postoperative NRS of 6.68 on the first postoperative day. Other surgeries on the foot, forefoot or ankle were among the top 15 of the most painful operations with a mean NRS value of at least 6 on the first postoperative day [2]. This highlights the importance of continuous regional anesthesia of the ankle and foot. In our study almost, all patients were pain-free on POD 0. Subsequently, all mean NRS values in both cohorts remained below 3.5 at all study time points, but only patients with self-coiling catheters were still below the generally accepted intervention threshold of NRS 3 with a mean NRS value of 2.7. The absence of ultrasound-guidance and confirmation of proper catheter location, varying use of local anesthetics and additional analgesics, heterogeneity of surgical procedures and the lack of information on mobilization strategies make comparability with other studies difficult. [23–25, 30–33]. Similar to our results, most studies show maximum pain intensity of NRS values up to 4 on POD 1 [23–25, 28–30] and a decreasing pain intensity int the further course.

### Oxycodone consumption

Patients in the self-coiling catheter group had lower oxycodone requirements in the first three days after surgery. While the lower consumption of oxycodone in the SCC group differed by 1.1 mg from the RSC group on the day of surgery, consumption was already 3.9 mg lower on POD 1. This difference in lower consumption in the SCC group reached significant levels at the day of surgery, POD 2 (4.9 mg) and POD 3 (3 mg), with an overall decrease in the need for additional opioids starting as early as the second postoperative day. Interestingly, the significant increased pain intensity in the RSC group did not reach the level that would have caused a difference for opioid consumption on POD 1.

Two facts might have influence on the accuracy of the discrimination. First, we used a single predefined dose of oxycodone 10 mg as rescue pain medication, which corresponded to our institutional multimodal pain concept. Therefore, discriminatory power regarding additional opioid need was low. Presumably, we could have more accurately assessed opioid need with a lower opioid bolus provided by patient controlled intravenous analgesia. Second, more than 50% of patients in the two groups had surgery that also involved the innervation of saphenous nerve. Despite reliable sciatic nerve blockade, the decaying effect of the single shot saphenous nerve blockade may have caused pain in the medial aspect to the ankle and foot and consequently led to increased opioid consumption at POD 1. Therefore, we could not distinguish, whether the opioid requirement was due to poor catheter performance or decreased or terminated saphenous nerve blockade. However, the number of saphenous nerve blocks performed did not differ between the two catheter groups. Overall, the amount of opioid consumption in the present study is consistent with previous studies investigating continuous sciatic nerve block during foot and ankle surgery [25, 28–30].

### Additional anesthesia procedures

Another aspect of the discussion is the possible influence of additional anesthetic procedures on postoperative pain intensity. Most patients (80.7%) underwent adjunct general anesthesia, while 11.4% received additional spinal anesthesia in addition to distal sciatic block. The remaining 7.9% received sedation or additional peripheral regional anesthesia. The choice of procedure was individually adapted to patients´ comorbidities and wishes. Due to randomisation and group size there were no significant differences in the distribution of additional anesthetic procedures between the two groups, so any potentially influence in this study is likely to be negligible. The question of whether spinal anesthesia has an influence on postoperative pain levels has not yet been conclusively determined. YaDeau et al. in a recent randomised controlled trial compared general and spinal anesthesia, each in combination with PNB for ankle and foot surgery. A significant difference in pain scores in favour of spinal anesthesia was found one hour after the end of the operation [31]. In contrast to long-acting morphine, we used fentanyl as an intrathecally supplemental opioid for spinal anesthesia. Therefore, an effect on pain intensity beyond the first 12 h seems unlikely.

### Catheter orifices

In the present study, we compared a self-coiling catheter with a closed tip and six lateral microholes with a conventional straight catheter having only a single orifice at the end. One might wonder whether this could have influenced our results. Fredrickson et al. studied the outflow of injected fluid on catheters with a different number of orifices. They showed a dependence of the fluid spread pattern on the fluid flow rate. At less than 80 ml per hour, the fluid left the catheters with multiple orifices only at the most proximal orifice [32]. Only at injection rate of more than 100 ml per hour were all microholes reached. Considering the flowrate of local anesthetics in our study of 6- 10 ml per hour, the self-coiling catheter probably functioned more like a catheter with one orifice. Therefore, we do not expect any relevant advantage of the multiple catheter orifice design in the present study. Furthermore, considering that the SCC was positioned only 2.4 cm into the target space within the perineural fascial sheath, with continuous infusion the local anesthetic may have left the most proximal orifice during continuous infusion, which only partly misses the target space when the catheter is withdrawn by muscle movements. Clinical data on the effect of catheter orifice design on the quality of pain management are inconsistent, although LA bolus application was used. It can be concluded that multiple orifice catheters, such as the SCC, function like conventional end-hole catheters at clinically relevant infusion rates. In this study, a maximum continuous flow rate of 10 ml per hour was achieved. Only bolus applications by the acute pain service were probably applied at rates above 100 ml per hour. Since this injection was performed manually from a 10 ml syringe, it is not possible to make precise statements about the application rate here [33, 34].

### Dislocation rate

Despite the widespread use of continuous regional anesthesia, the issue of perineural catheter dislocation is not well elucidated in studies nor in clinical practice. Therefore, the results of our study provide new insights into factors contributing to catheter dislocation. To our best knowledge, there is no clinical study investigating the dislocation rate of self-coiling catheters compared to regular catheters. Luyet et al. had shown a lower initial dislocation rate for self-coiling catheters in human cadavers. However, the design of the cadaver study did not address dislocation rates in the further course [19].

Significantly fewer self-coiling catheters (14%) slipped out of the subparaneural target space during the study period than conventional catheters with straight ends (60%). This significant difference could be due to different insertion distances within the perineural fascia sheath. Accordingly, Ilfeld et al. [35] and Steffel et al. [16] described a higher dislocation rate with a smaller insertion distance at the nerve. In the control group, we used a regular straight catheter with a removable metal wire. Such firm catheters often pass through the target structure and protrude about 3 cm beyond the needle tip during initial insertion and thus also out of the perineural fascia sheath, especially when the catheters are advanced in-plane perpendicular to the SAX-imaged nerve. Therefore, we had to adjust the catheter by retraction until the LA injection was well distributed within the perineural fascia sheath under sonographic view. The self-coiling catheter provide a more reliable initial placement without passing through the target space when the insertion distance of 3 cm beyond the needle tip is not exceeded with an in-plane approach [19]. Thus, self-coiling catheters are less likely to require retraction due to initial misplacement and a longer catheter segment remains around the target structure.

Postoperative breakthrough pain and the need for additional systemic analgesics are often considered surrogate markers of inadequate catheter performance, regardless of the reason. However, this concept is misleading in evaluating improper catheter position. For postoperative assessment of catheter position and initial confirmation of correct catheter placement, we used sonographic imaging of the saline bolus via the catheter. Although the efficacy of continuous regional anesthesia depends on correct catheter placement, assessment of catheter position is still not a common procedure neither in studies nor in clinical practice [36]. However, it is important to remember here that visualization of catheter position is often compromised by sterile dressings and swollen tissue in the affected area.

In our study, most dislocations were detected after arrival in the PACU. It can be assumed that passive and/or active movement of the thigh muscles occur when the patient is positioned before surgery or during patient transfer to bed. This causes a mechanical traction on the catheter in the biceps femoris muscle, which withdraws the catheter from the target space within the nerve-surrounding fascia.

Marhofer et al. studied within-tissue dislocation rates in healthy volunteers. They found a lower dislocation rate after motion of 25% and 5% for perineural femoral and interscalene catheters, respectively. Only regular straight catheters were used in this study [15]. In contrast to our study, catheters were examined for only six hours after placement. In addition, both catheters were placed using the out-of-plane technique, which is more robust against dislocation [10].

Only two studies address the rate of internal dislocation rates during continuous popliteal sciatic nerve block. Steffel et al. compared a catheter-over-needle (CON) with a conventional catheter-through-needle (CTN) technique in human cadaver [16]. Twenty-seven percent of CON catheters dislodged from the fascial sheath of the sciatic nerve. In contrast, all conventional catheters CTN remained perineural. Remarkably, Steffel et al. did not evaluate the spread of the local anesthetic, but only visualized sonographically the presumed end of the catheter by sonography. However, the spread of LA around the target is the key determinant of adequate nerve block. Comparisons with our study are difficult because it involved only a small cohort of 30 subjects and the tissue characteristics of the body donors cannot be considered identical to those of living subjects [16]. In contrast to our patients, only passive flexion movements were performed on the body donors. However, we believe that active contraction and relaxing of the muscle contributes significantly to the movements of the catheter in the tissue. Hauritz and colleagues compared two different approaches to popliteal sciatic blockade in terms of dislocation in the tissue. In contrast to our patients, they confirmed the position of the catheter 48 h after application using an MRI contrast bolus [10]. While a dislocation rate of 10% was reported for the out-of-plane approach, the use of an in-plane technique similar to our study protocol resulted in a significant higher dislocation rate of 40%. In our study, an even higher dislocation rate of 60% was found for conventional catheters. An important difference is the longer part of the catheter that remains under the perineural fascia sheath. A catheter distance within the perineural fascia sheath of 1.6 cm in our study compared to 3.4 cm in the investigation of Hauritz et al. may increase catheter dislocations due to traction forces, as mentioned above. Interestingly, the dislocation rate decreased when a self-coiling catheter was used with the in-plane approach to a level reported by Hauritz et al. for the out-of-plane technique. Regular straight perineural catheters appear to be more reliable in terms of dislocation rate when using out-of-plane approach, whereas the self-coiling catheters can be placed with good results when using the in-plane technique.

Evidence suggests that the site of perineural catheter injection relative to the bifurcation of the sciatic nerve may influence the quality of analgesia [37]. However, in the study by Monahan et al. there was no control of the position of the catheter tip in the postoperative course. In our study, as expected, fluid spread occurred at the same level of bifurcation as placement. The curled catheters roll up at the initial advancement site with a fixed radius of approximately 0.5 cm and therefore do not tend to dislocate significantly secondarily cranially or caudally. The regular catheter is more likely to dislocate secondarily because of its stiffness in the direction of needle insertion, usually toward the insertion site. Several methods for visualizing catheter position have been described in the literature, e.g.,visualization of injected fluids (saline, LA, contrast medium) or air or direct visualization of the catheter [14, 16, 38–40]. Imaging techniques include high resolution ultrasound (HRUS), computer tomography (CT), or magnetic resonance imaging (MRI) [10, 19, 38, 39]. There are advantages and disadvantages for each technique, which we would like to discuss briefly. We have chosen saline injection to check the position of the catheter tip because it is a safe and straightforward method that is part of our daily clinical routine. The use of air or agitated fluid with microbubbles can improve the contrast and visibility of the injectate. However, the propagation of air in the tissue significantly degrades the quality of sonographic visualization of the target structure and surrounding tissue by scattered ultrasound waves. In contrast, visualization of saline via the catheter is similar to the common procedure for observing the injection of LA via the cannula. Finally, direct visualization of the catheter is less favourable. On the one hand, despite their improved echogenicity, catheters can hardly be aligned with the ultrasound plane like a firm needle. This issue is aggravated by the use of self-coiling catheters. On the other hand, controling the spread of the LA around the target structure is more important for reliable blocking success than the position of the catheter tip itself. Compared to CT and MRI, HRUS imaging is widely available and a real point of care technique that can be applied as often as desired. Also, patient transports and staff expenses will be avoided. In addition, patients are exposed to radiation during CT scan.

### Leakage and catheter shift at the insertion site

The maximum leak rate was 31% in both groups in our study, identical to the leak rate of 31% for the CTN technique in another study [38]. Lower leak rates of 13.9% have been reported for continuous distal sciatic catheters [41]. Leakage problems are common with catheter-through-needle access, because needle´s diameter is larger than that of the catheter. As a result, the tissue does not seal the puncture track along the catheter, and the injected fluid and interstitial fluid or blood may flow retrogradely. Although the catheter-over-needle technique reduces the occurrence of leakage, clinical studies have not yet demonstrated superiority in terms of dislocation rates [38, 42, 43]. Accordingly, we could not find a correlation between leakage and dislocations, as both study groups had the same leakage rate. Dislocation rates in terms of the catheter slipping out of the skin at the insertion site are reported to have an incidence of 0.5 to 26% [8, 9]. In our study we considered slip out at the insertion site of 1 cm or more as clinically relevant movement. This was the case in 10% in SCC group and 12.6% in RSC group. According to findings of Marhofer et al. we could not observe a significant influence of outwardly slipping catheters at insertion site on the dislocation rate in the target area [15].

### Complications

The overall complication rate was very low. No persistent neurologic deficit was observed. The most serious complication was a patient who experienced a metallic taste several hours after catheter application, which was considered as a mild systemic toxicity sign of the local anesthetic. The ropivacaine infusion was stopped. Neither a negative aspiration test nor sonographic imaging of fluid spread via the catheter revealed secondary intravascular dislocation. The catheter was immediately removed, whereupon symptoms resolved completely. Any redness of the insertion site during the daily visit was considered as mild infection. The catheters were removed immediately without any further sequels. The overall rate of mild infection of 5.7% observed here is within the results of other studies [44, 45].

In this study, catheter occlusion occurred in one patient from each cohort (total 1.4%). Ma et al. reported this rare incident at a rate of 1% [43].

### Limitations

Our study results are limited by the use of certain drugs. Thus, while the dislocated catheters would likely have been detected earlier if a short- or medium-long acting local anesthetic had been used as an alternative to ropivacaine for the initial bolus. In addition, for rescue pain management, patients received opioids only as needed at a fixed dosage of 10 mg oxycodone orally by the nursing staff. A more finely tuned opioid application, e.g., by patient-controlled intravenous analgesia could have more clearly reflected the actual need for additional analgesics. Another limitation is the concept of single blockade of the saphenous nerve with limited pain relief of 12 to 18 h. Thus, NRS values may have been influenced by pain perception in saphenous nerve innervation area. Furthermore, the design of our study was not double blinded because the ultrasonographer could occasionally draw conclusions about catheter type by observing insertion depth and specific catheter length designations. The results of this study apply only to our chosen approach via the short axis view of the nerve and the in-plane needle approach. Beyond that, our results are not transferable to other catheter designs or alternative insertion sites. In the case of sonographically confirmed catheter dislocation in situ, no further positional checks were performed. Whether it is possible that the malposition could spontaneously transform into a renewed perineural position remains unclear. Moreover, extrafascial dislocation does not necessarily imply a complete loss of efficacy. Our continuously applied ropivacaine may still have been sufficient to relieve pain by spreading the local anesthetic toward the nerve along the residual puncture pathway or by diffusion through the connective tissue. Based on experience with the efficacy of interfascial plane blockades (e.g. the erector spinae blockade), we suspect some analgesic effect of even small amounts of LA by blockade of small C-fibres, even if the site of application is not near the target nerve.

## Conclusions

The self-coiling catheter design for continuous blockade of the sciatic nerve near the popliteal fossa provides better postoperative pain control and a lower dislocation rate in the tissue than a conventional straight catheter design. Secondary migration away from the target structure, could only be visualised sonographically and was unrelated to the external appearance of the puncture site. Further studies for other alternative techniques and localization are needed to further evaluate this catheter design.

## Data Availability

The datasets used and/or analysed during the current study are available from the corresponding author on reasonable request.

## Abbreviations

AE: Adverse event
CTN: Catheter-through-needle
CON: Catheter-over-needle
CONSORT: Consolidated Standards of Reporting Trials
IP: In-plane
LA: Local anesthetic
NRS: Numeric rating scale
OOP: Out-of-plane
PACU: Post anesthesia care unit
PNB: Peripheral nerve block
POD: Postoperative day
RA: Regional anesthesia
RSC: Regular straight catheter
SAE: Severe adverse event
SAX: Short axis view
SCC: Self-coiling catheter
US: Ultrasound
